# Gait Biomechanics of Individuals with Transtibial Amputation: Effect of Suspension System

**DOI:** 10.1371/journal.pone.0096988

**Published:** 2014-05-27

**Authors:** Arezoo Eshraghi, Noor Azuan Abu Osman, Mohammad Karimi, Hossein Gholizadeh, Ehsan Soodmand, Wan Abu Bakar Wan Abas

**Affiliations:** 1 Prosthetist & Orthotist, Department of Biomedical Engineering, Faculty of Engineering, University of Malaya, Kuala Lumpur, Malaysia; 2 Department of Biomedical Engineering, Faculty of Engineering, University of Malaya, Kuala Lumpur, Malaysia; 3 Department of Orthotics & Prosthetics, Isfahan University of Medical Sciences, Isfahan, Iran; 4 Department of Human Locomotion, Technische Universität Chemnitz, Chemnitz, Germany; University of Toronto, Italy

## Abstract

Prosthetic suspension system is an important component of lower limb prostheses. Suspension efficiency can be best evaluated during one of the vital activities of daily living, i.e. walking. A new magnetic prosthetic suspension system has been developed, but its effects on gait biomechanics have not been studied. This study aimed to explore the effect of suspension type on kinetic and kinematic gait parameters during level walking with the new suspension system as well as two other commonly used systems (the Seal-In and pin/lock). Thirteen persons with transtibial amputation participated in this study. A Vicon motion system (six cameras, two force platforms) was utilized to obtain gait kinetic and kinematic variables, as well as pistoning within the prosthetic socket. The gait deviation index was also calculated based on the kinematic data. The findings indicated significant difference in the pistoning values among the three suspension systems. The Seal-In system resulted in the least pistoning compared with the other two systems. Several kinetic and kinematic variables were also affected by the suspension type. The ground reaction force data showed that lower load was applied to the limb joints with the magnetic suspension system compared with the pin/lock suspension. The gait deviation index showed significant deviation from the normal with all the systems, but the systems did not differ significantly. Main significant effects of the suspension type were seen in the GRF (vertical and fore-aft), knee and ankle angles. The new magnetic suspension system showed comparable effects in the remaining kinetic and kinematic gait parameters to the other studied systems. This study may have implications on the selection of suspension systems for transtibial prostheses.

**Trial Registration:**

Iranian Registry of Clinical Trials IRCT2013061813706N1.

## Introduction

The primary goal of rehabilitation of lower limb amputees is to resume normal gait as much as possible. Prosthetic devices should allow normal gait function using the most appropriate components. Gait asymmetry is one of the main concerns in unilateral lower limb amputees to avoid exertion of excessive load on the sound limb [1], [2]. Previous research findings have been controversial over the kinetic and kinematic differences between the amputated and sound legs. Several studies indicated higher reliance on the sound leg by increased loading and stance time, which has been attributed to ankle loss in transtibial amputees [3], [4]. On the other hand, some literature supported the idea that amputees may not need to rely on the intact leg owing to the compensatory mechanisms adopted by the amputated leg [5]. Winter and Sienko (1988) explained that the amputee-related literature increasingly refers to variables that measure gait symmetry [6]. Therefore, a scientific justification is needed to encourage more symmetrical walking pattern.

The influence of various prosthetic components on the gait of lower limb amputees has been evaluated. Extensive research has been conducted on the effects of prosthetic foot as transtibial amputees lose normal ankle mechanics while retain the anatomical knee joint [7]–[10]. Moreover, the improper fit of the prosthetic socket and failure of the suspension system can result in pistoning, which in turn will affect the walking pattern. Total surface bearing (TSB) socket was introduced as new concept, and its total contact was said to eliminate pistoning during walking [11]–[14]. Researchers have also studied the effects of prosthetic liner on the gait of transtibial amputees and revealed that liner thickness can affect the gait variables [15].

Current suspension systems for transtibial amputees are either pin/lock or seal liners, which are both provided with TSB sockets. Suspension systems have been investigated in terms of interface pressure, interface dynamics (pistoning) and comfort. Pin/lock systems are said to cause pain and discomfort inside the prosthetic socket, leading to skin changes in the long term. Discomfort may cause changes in gait parameters as the amputee would be reluctant to bear load over the prosthetic socket during walking. The Seal-In suspension liner can relieve the distal end pressure by applying more loads to the proximal tissues of the residual limb. Both systems control pistoning, but the Seal-In liner is more successful. These two suspension types have not been studied in terms of gait parameters during level walking.

A new magnetic prosthetic suspension system (MPSS) has been introduced, and compared with the pin/lock and Seal-In liners in terms of pistoning through gait simulation, as well as interface pressure [16], [17]. This hypothesis-generating study aimed to examine the changes in gait characteristics of transtibial amputees with the MPSS, pin/lock and Seal-In suspension systems. We were interested to find out what gait parameters show significant changes. It was also intended to see how deviated was the gait pattern with every suspension type from the gait of normal individuals. The main hypothesis of this study was that the type of suspension may significantly alter the kinetic and kinematic gait parameters as well as pistoning. Furthermore, it was assumed that the sound and prosthetic legs would exhibit significantly different patterns.

## Materials and Methods

The protocol for this trial is available as supporting information; see Protocol S1.

### Ethics Statement

The ethics committee of the University of Malaya Medical Center approved the study. The subjects signed consent forms prior to participation.

### Methods

In a clinical trial, fifteen individuals with transtibial amputation were selected to participate in the study as sample of convenience. Amputees were eligible for the study if they were unilateral transtibial, could ambulate independently, had a stump free of ulcer and pain, had undergone amputation at least one year prior to the study, and had healthy upper limbs to don and doff the prosthesis without help. The subject recruitment was performed from March 2012 to March 2013.

**Figure 1:**
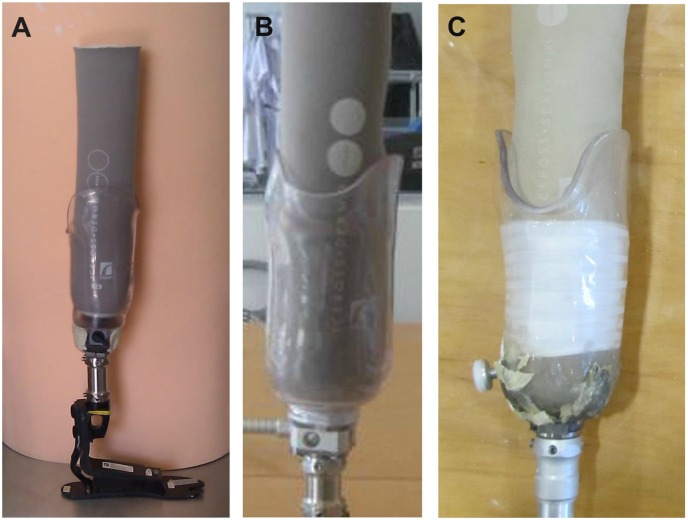
The suspension systems used in this study. A) MPSS; B) Pin/lock and C) Seal-In suspension systems.

Inconsistency of the prosthetic fabrication techniques, alignment, and fitting can significantly influence the outcome. Therefore, one of the authors (a registered prosthetist) fabricated three prosthetic systems for each participant. The only difference between the prostheses was the suspension system. The suspension systems were: a) pin/lock suspension (Dermo liner with shuttle lock), b) new magnetic lock (MPSS), and c) Seal-In system (Seal-In X5 liner) (Figure 1). The third system required a separate negative cast; whereas the first two systems were fabricated from a single negative cast. The prosthetist ensured the fit of each prosthetic socket through a transparent check socket (Northplex, North Sea Plastic Ltd) while standing in the alignment frame and during walking. The sockets were required to be TSB; therefore, the transparent material allowed close inspection of fit.

The characteristics of the new prosthetic suspension system have been described elsewhere [17]. In brief, the new system was designed to be used with silicone liners as they are commonly used. To this end, a cap was designed that matched both the main body of the new coupling device, and the liner’s distal end. The dimensions were purposely designed to match the liner proportions. A central screw enabled coupling to the liner. The body of the coupling device was source of magnetic power. As such, the cap was made of mild steel to produce high gripping force. A permanent magnet was utilized that was capable of generating a strong magnetic power. The housing intensified the magnetic field by flanges. In order to control the magnetic power, a mechanical switch was affixed to the housing and the magnet. When the rotary switch was in the “On” position, the cap was attracted to the housing, whereas it was released from the lower body of the coupling device when the switch was in the “Off” position.

Pyramid adapters connected the TSB sockets to the aluminum alloy pylon and prosthetic foot (Flex-foot Talux, Ossur). The subjects were also provided with three definitive sockets for the acclimation period of four weeks. The aligning procedure was performed using a laser liner to ensure accuracy. The subjects were trained for walking with the new prosthetic legs as follows. After ensuring the fit of prosthetic sockets, the training prostheses were fabricated. Every participant was required to attend the Brace & Limb Laboratory, University of Malaya for the gait training during one week. The gait training was performed in the parallel bars to check the dynamic alignment during level walking. Next, the amputees participated in training out of the parallel bars, climbing the stairs and ramp in real environment. Necessary adjustments were applied so that the participants were fully confident to ambulate without pain or discomfort. The subjects used identical shoes in all the experiments.

A Vicon motion analysis system (612 Oxford Metrics; Oxford, UK) with six cameras (MXF20) was utilized to evaluate the gait kinematics and pistoning between the prosthetic socket and liners. Kinetic data was recorded using two Kistler force platforms (type 28112A2-3S, Kistler Holding AG, Switzerland). The synchronized frequency was set at 200 Hz. For the pistoning measurement, the authors introduced a new measurement technique using the Vicon motion system [18]; the same method was adopted in this study. The location of the ankle reflective marker on the prosthetic foot approximated the axis of rotation for the sound ankle. The subjects walked with each prosthesis type adopting self-selected speed on a 10-meter level walkway. Five successful trials were selected for the kinetic and kinematic analyses. A trial was considered as appropriate if both feet landed properly on the force plates (whole foot was on the force plate). The participants could rest between the trials. All data was collected at the motion laboratory of Center for Applied Biomechanics, University of Malaya. Butterworth filter with a cutoff frequency of 10 Hz was used to filter the data.

### Data Analysis

Kinematic and kinetic gait parameters were processed using the Vicon Nexus (Oxford Metrics, Ltd.) software. Data was analyzed based on the percentage of gait cycle. The average values of the five trials were used for the analysis. Statistical analyses were performed using SPSS 18.0. The normality of variables was verified by the Kolmogorov-Smirnov test. The one-way Repeated Measures Analysis of Variance (ANOVA) with the Bonferroni test was used to compare the three suspension systems. The paired samples t test was adopted to compare between the sound and prosthetic legs. In comparisons among the suspension systems, only the prosthetic limb was considered. The level of significance was set at 0.05. The Cohen’s *d* of 0.2 to 0.3 might show a “small” effect, around 0.5 is a “medium” effect and 0.8 to infinity may be considered a “large”n effect. The pistoning was measured during the stance and swing phases of gait. The parameter values were averaged over 5 trials, not over the suspension systems. That is, every individual was tested separately with each of the suspension systems, which is considered as repeated measure. Additionally, each testing procedure with each suspension system was repeated for 5 times. Then, the average score of 5 trials with each system was separately used in the repeated measures ANOVA.

The following kinetic and kinematic gait parameters were evaluated: step length, walking speed, stance and swing time (percentage), vertical ground reaction force (GRF), fore-and-aft GRF hip, knee and ankle angles. The step cycle for both legs started with the heel strike. Data for each time frame were normalized to the whole stride time due to the variability in walking speed [19]. Furthermore, the fore-aft and vertical GRF were normalized to the body weight.

The gait deviation index (GDI) was also calculated for each system. The electronic template of the developers was used to calculate the GDI [21]. This template compares the input data with a database of 166 normal subjects. The measures were calculated for the prosthetic limbs of every subject and for each suspension system. The sound limb may exhibit higher kinematic deviations than the prosthetic limb because of the compensatory mechanisms. Thus, the average data for every gait summary measure was used to generate a one-dimensional gait deviation measure.

GDI calculation necessitated a matrix of healthy control data. In brief, the data comprised rows of kinematic data at 2% increments of the gait cycle (459 datum = 9 angles 51 points), as well as columns of data from different subjects [21]. Kinematic data included ankle dorsi/plantarflexion, knee flex/extension, hip and pelvic angles in all three planes, and foot progression.

The GDI for amputee subject α based on the distance between the normal control (TD) and the amputee subject was calculated from the following equation [21]:
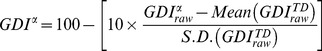
(1)As GDI determines the distance from the mean normal gait, GDI of 100 or greater shows that gait pathology is absent. With every deviation of 10 points from 100, the gait is one standard deviation away from the normal. For instance, if GDI^α^ = 55, the gait of subject α is 4.5 standard deviation away from the normal.

## Results

From the 15 participants, only the data for thirteen individuals were included in the statistical analysis. The protocol required the subjects to participate in several casting, fitting and training sessions for 3 different prosthesis types in addition to the experiment sessions. Two subjects did not manage to complete the sessions due to their job limitations and were excluded from the study. The individual characteristics ants are shown in Table 1.

**Table 1:** Characteristics of the participants.

Subject no.	Age	Height (cm)	Mass (Kg)	Amputated side	Cause of amputation
1	42	173	75	Left	Diabetes
2	37	168	90	Left	Trauma
3	30	182	60	Left	Trauma
4	72	166	75	Left	Diabetes
5	46	167	64	Left	Trauma
6	35	170	99	Left	Diabetes
7	49	164	57	Right	Diabetes
8	53	177	60	Right	Diabetes
9	41	167	66	Right	Trauma
10	33	162	94	Left	Trauma
11	26	170	79	Left	Trauma
12	60	176	83	Right	Diabetes
13	59	169	75	Right	Diabetes

### Pistoning

The repeated measures ANOVA indicated significant differences among the three studied suspension systems during gait (*F*(2,24) = 27.81, *P* = 0.000 and η_p_^2^ = 0.70). In the swing phase, *F*(2,24) = 46.49, *P* = 0.000 and η_p_^2^ = 0.79, while it was *F*(2,24) = 27.13, *P* = 0.000 and η_p_^2^ = 0.69 during stance. Overall, the magnitude of pistoning with the Seal-In suspension was considerably lower compared with the pin/lock and MPSS during swing (*P = *0.000 and *P = *0.001, respectively).

Comparisons between the MPSS and Seal-In systems revealed higher vertical displacements (piston motion) when the prosthetic limb was suspended using the MPSS (*P = *0.001). This significantly higher pistoning was evident during the swing phase; yet, the magnitudes of pistoning were higher for the Seal-In liner during the stance (*P = *0.000).

Statistical analyses indicated lower pistoning values with the MPSS compared with the pin/lock system during the swing phase (*P* = 0.035). During one gait cycle, 4.06 mm and 2.88 mm of pistoning was observed with the pin/lock and MPSS (*P* = 0.019).

### Kinetics and Kinematics

The suspension type did not alter the walking speed, stance and swing time significantly (*P*>0.05). The swing time of the prosthetic side were significantly longer than the sound limb with the three suspension systems (*P*<0.05) (Table 2). However, the stance time was significantly lower on the prosthetic limb than the sound limb. Significant differences were found between the suspension systems in the first peak of vertical GRF (loading response) (*F*(2,24) = 13.01, *P* = 0.000, η_p_^2^ = 0.52). The comparison between the MPSS and pin/lock as well as the Seal-In and pin/lock revealed significant differences (*P* = 0.042 & *P = *0.006, respectively). With all three systems, weight transfer during the transition from double- to single-limb support occurred in a shorter period for the sound leg compared with the prosthetic leg (Table 2).

**Table 2:** Kinetic and kinematic differences between the sound and prosthetic limbs within every suspension type; Mean (95% CI).

Parameters	Seal-In		*P* value	MD (CI)	*d*	Pin/lock		*P* value	MD (CI)	*d*	MPSS		*P* value	MD (CI)	*d*
	Sound	Prosthesis				Sound	Prosthesis				Sound	Prosthesis			
Step length (m)	0.57 (0.53–0.61)	0.61 (0.55–0.66)	0.320	0.04 (−0.84–3.09)	0.1	0.54 (0.47–0.62)	0.62 (0.54–0.69)	0.134	0.08 (−0.03–0.17)	0.5	0.56 (0.5–0.62)	0.59 (0.51–0.67)	0.536	0.03 (−0.07–0.12)	0.2
Cadence (step/min)	94.09 (92.73–95.46)	95.21 (94.02–96.41)	0.183	1.12 (−0.85–3.09)	0.2	93.03 (91.77–94.3)	95.60 (94.13–97.25)	**0.031**	2.57 (0.28–4.03)	0.4	93.03 (91.77–94.3)	95.06 (93.37–96.75)	0.145	2.03 (−0.73–4.4)	0.6
Stance time (% of gait cycle)	65.56 (64.1–67.03)	62.28 (60.89–63.70)	**0.002**	3.28 (−5.11–−1.45)	1.3	66.7 (65.53–67.87)	60.73 (59.74–61.73)	**<0.001**	5.97 (−7.38–−4.55)	3.4	65.57 (64.34–66.8)	62.31 (61.19–63.42)	**0.001**	3.26 (−4.77–−1.75)	1.7
Swing time (% of gait cycle)	34.46 (33.31–35.61)	37.70 (65.60–67.80)	**0.001**	32.24 (30.74–33.75)	1.4	33.32 (31.64–35)	38.30 (36.95–39.65)	**<0.001**	4.98 (3.32–6.64)	2.1	34.14 (32.75–35.52)	37.56 (36.39–38.73)	**0.001**	3.42 (1.83–5.02)	1.7
Vertical GRF, 1^st^ peak (%BW)	121.11 (118.05–124.17)	99.68 (97.15–102.22)	**<0.001**	21.43 (−25.15–−17.7)	4.8	126.68 (123.88–129.48)	104.22 (101.58–106.87)	**<0.001**	22.46 (−26.03–−18.89)	4.9	115.27 (109.13–121.42)	96.42 (91.84–101.02)	**<0.001**	18.85 (−25.2–−12.49)	2.3
Vertical GRF, 2^nd^ peak (%BW)	101.99 (99.59–104.4)	102.63 (100.19–105.06)	0.706	0.64 (−1.69–2.96)	0.1	101.12 (98.87–103.38)	99.09 (96.34–101.85)	0.301	2.03 (−6.12–2.06)	0.4	105.18 (102.38–107.98)	91.69 (88.51–94.87)	**<0.001**	13.49 (−17.49–−9.49)	2.4
Fore-aft GRF, 1^st^ peak (%BW)	7.86 (7.1–8.62)	5.45 (4.79–6.12)	**<0.001**	2.41 (−3.34–−1.47)	2.1	9.34 (8.4–10.28)	4.66 (3.98–5.35)	**<0.001**	4.68 (−5.7––3.66)	3.9	9.86 (8.94–10.78)	4.11 (3.43–4.80)	**<0.001**	5.75 (−6.87–−4.61)	4.8
Fore-aft GRF, 2^nd^ peak (%BW)	−7.51 (−8.25–−6.77)	−8.10 (–8.76– −7.43)	0.208	0.59 (−1.37–0.19)	0.5	−7.13 (−8.84– −6.45)	−8.11 (−8.91– −7.31)	0.058	0.98 (−2.04–0.04)	0.7	−7.01 (−8.10– −6.25)	−7.41 (−8.13– −6.69)	0.390	0.40 (−1.25–0.52)	0.3
Hip position-initial contact	35.89 (33.81–37.97)	32.8 (30.95–34.65)	0.193	3.09 (−5.38–−0.8)	0.9	32.6 (30.94–34.26)	33.11 (31.04–35.17)	0.543	0.51 (−1.26–2.27)	0.2	34.15 (32.11–35.81)	33.04 (31.08–35.00)	0.318	1.11 (−3.44–1.21)	0.4
Max Hip Ext	−2.13 (−2.46–−1.81)	3.06 (2.71–3.42)	**<0.001**	5.19 (4.83–5.56)	3.6	−2.42 (−2.98–−1.85)	2.62 (2.18–3.05)	**<0.001**	5.04 (4.36–5.71)	3.6	−2.42 (−2.75– −1.67)	2.5 (1.97–3.04)	**<0.001**	4.92 (4.29–5.53)	5.4
Hip ROM	38.42 (37.37–39.47)	37.31 (35.83–38.79)	0.193	1.11 (−2.66–0.43)	0.5	37.23 (35.03–38.80)	36.13 (34.92–37.33)	0.121	1.1 (−2.55–0.34)	0.5	37.52 (35.67–39.45)	36.7 (35.25–38.16)	0.261	0.82 (−2.18–0.65)	0.4
Knee position-initial contact	1.41 (1.14–1.67)	5.4 (4.55–6.25)	**<0.001**	3.99 (3.12–4.87)	3.8	4.1 (3.17–5.02)	5.73 (4.9–6.57)	**0.022**	1.63 (0.28–2.99)	1.1	3.9 (3.35–4.45)	5.53 (4.34–6.71)	**0.023**	1.63 (0.27–2.98)	1.1
Max Knee Flex-stance	15.12 (14.09–16.15)	13.72 (12.59–14.86)	0.059	1.40 (−2.98–0.18)	0.8	13.43 (11.86–15.01)	12.47 (11.08–13.85)	0.302	0.96 (−2.93–0.99)	0.4	14.24 (12.66–15.82)	12.84 (11.5–14.19)	0.235	1.40 (−3.83–1.04)	0.6
Max Knee Flex-swing	55.17 (53.58–56.75)	75.40 (73.21–77.57)	**<0.001**	20.23 (17.32–23.13)	6.4	52.52 (51.08–53.96)	66.92 (64.77–69.08)	**<0.001**	14.4 (11.49–17.32)	4.7	54.02 (52.06–55.97)	70.81 (68.7–72.93)	**<0.001**	16.79 (14.21–19.38)	5.0
Knee ROM	56.14 (54.57–57.7)	70.68 (68.34–73.04)	**<0.001**	14.54 (11.54–17.57)	4.4	52.61 (51.12–54.09)	61.42 (58.99–63.81)	**<0.001**	8.81 (6.28–11.31)	2.7	52.79 (51.28–54.3)	58.25 (56.55–59.94)	**<0.001**	5.46 (3.02–7.89)	2.1
Ankle position-initial contact	2.12 (1.59–2.65)	−0.81 (−1.21– −0.41)	**<0.001**	2.93 (−3.67–−2.19)	3.8	–4.21 (−4.88–−3.54)	0.27 (0.07–0.46)	**<0.001**	4.48 (3.76–5.19)	5.5	−2.29 (−2.81–−1.77)	−0.6 (−0.93– −0.28)	**<0.001**	1.69 (1.01–2.37)	2.3
Max ankle PF-stance	−6.68 (−8.33–−5.02)	−7.19 (−8.3– −6.07)	0.583	0.51 (−2.75–1.73)	0.2	−5.92 (−7.23–−4.62)	−5.89 (−6.98– −4.81)	0.951	0.03 (−1.09–1.15)	0.0	−6.12 (−7.41–−4.82)	−3.02 (−3.73– −2.31)	**0.002**	3.10 (1.42–4.77)	1.8
Max ankle DF-stance	7.3 (6.23–8.37)	14.49 (13.34–15.63)	**<0.001**	7.19 (5.44–8.93)	3.9	8.09 (7.07–9.1)	15.11 (14.24–15.98)	**<0.001**	7.02 (5.72–8.32)	4.5	7.92 (6.78–9.06)	14.67 (13.93–15.41)	**<0.001**	6.75 (5.43–8.06)	4.2
Max ankle PF-swing	−13.2 (−14.7–−11.7)	0.33 (0.12–0.55)	**<0.001**	13.53 (12.02–15.05)	7.6	−12.15 (−13.2–−11.1)	1.37 (1.13–1.67)	**<0.001**	13.52 (12.45–14.64)	5.7	−12.17 (−13.05–−11.29)	1.13 (0.93–1.33)	**<0.001**	13.30 (12.38–14.21)	5.2
Ankle ROM	20.67 (19.1–22.24)	21.73 (20.35–23.1)	0.280	1.06 (−1.23–3.35)	0.4	20.08 (18.68–21.48)	20.87 (19.32–22.43)	0.508	0.79 (−1.74–3.33)	0.3	20.25 (18.5–21.99)	20.69 (19.55–21.83)	0.700	0.44 (−2.00–2.88)	0.2

CI = Confidence interval; PF = plantar flexion; DF = dorsiflexion; Flex = flexion; Ext = extension; ROM = range of motion; MD = mean difference.

*Values of significance (*P*<0.05) have been shown in bold.

*d* equals to values of Cohen’s d; 0.2 = small, 0.5 = medium, >0.8 = large.

The vertical GRF during the loading response (2^nd^ peak) was significantly different among the three systems (*F*(2,24) = 18.80, *P* = 0.000, η_p_^2^ = 0.79). None of the systems showed significant difference between the sound and prosthetic leg. From the double- to single-limb support (swing time), the weight shift occurred at a considerably shorter period for the sound limb compared with the prosthetic limb for all the systems (all *P* = 0.000).

The suspension systems not only changed the first peak of the fore-aft GRF significantly (*F*(2,24) = 14.57, *P* = 0.003, η_p_^2^ = 0.65), but also there was significant difference between the sound and prosthetic legs within every suspension type (all Cohen’s *d*>0.8). The magnitudes of 1^st^ peak fore-aft GRF were significantly lower on the prosthetic leg compared with the sound leg for all the systems (all *P* = 0.000, *d*>0.8) (Table 2). The lowest mean difference was seen with the Seal-In system (2.40).

The average knee range of motion (ROM) was significantly different among the three studied systems (*F(*2,24) = 46.48, *P* = 0.000, η_p_^2^ = 0.79). The highest knee ROM with the prosthetic leg was seen with the Seal-In (70.7°). There was no significant difference between the pin/lock and MPSS (*P* = 0.075). The knee ROM was significantly different between the legs for the Seal-In, pin/lock and MPSS (*P* = 0.000; *d* = 4.4, *d* = 2.7, *d* = 2.1, respectively). A significant difference was observed among the three systems in the maximum knee flexion (*F*(2,48) = 18.40, *P* = 0.000, η_p_^2^ = 0.60). The highest knee flexion was seen with the Seal-In, followed by the MPSS and pin/lock (*P* = 0.006 & 0.001, respectively).

**Figure 2:**
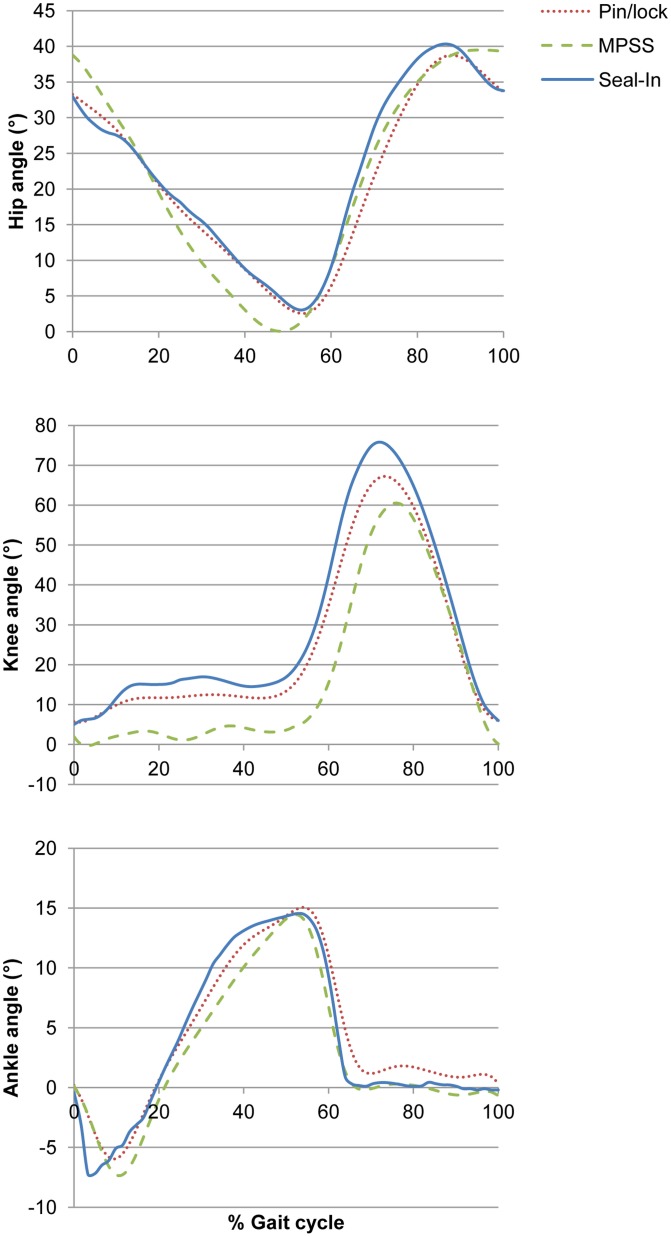
Kinematic values based on the suspension type. Comparison of kinematic values for prosthetic limbs among the different suspension systems (n = 13).

Tables 2 and 3 show the mean values, confidence intervals and effect sizes of kinetic and kinematic gait parameters based on the suspension type. Figure 2 illustrates the comparison of kinematic values among the suspension systems for the prosthetic limb.

**Table 3:** Comparison of kinetics and kinematic variables with regards to the suspension system type in the prosthetic limb.

Parameter	Suspension type			*P* value	Effect size
	Mean (95% CI)				
	Seal-In	Pin/lock	MPSS		
Step length (m)	0.61	0.62	0.60	0.817	0.03
	(0.55–0.66)	(0.54–0.69)	(0.51–0.67)		
Cadence (step/min)	95.2	95.70	95.06	0.844	0.14
	(94.02–96.41)	(94.13–97.25)	(93.37–96.75)		
Velocity (m/s)	0.94	0.91	0.98	0.075	0.23
	(0.91–0.98)	(0.86–0.96)	(0.95–1.01)		
Stride length (m)	1.21	1.12	1.08	0.118	0.16
	(1.14–1.29)	(1.03–1.20)	(0.95–1.22)		
Stance time (% of gait cycle)	62.28	61.73	62.50	0.062	0.39
	(60.89–63.70)	(59.74–61.73)	(61.19–63.42)		
Swing time (% of gait cycle)	37.70	38.30	37.56	0.435	0.06
	(65.60–67.80)	(36.95–39.65)	(36.39–38.73)		
Vertical GRF, 1st peak (%BW)	99.68	104.22^a^ ^,c^	96.42^b^	**<0.001** *	0.52
	(97.15–102.22)	(101.58–106.87)	(91.84–101.02)		
Vertical GRF, 2nd peak (%BW)	102.63	99.09	91.69^a^^,^ b	**<0.001** *	0.61
	(100.19–105.06)	(96.34–101.85)	(88.51–94.87)		
Fore-aft GRF, 1st peak (%BW)	5.45	4.66^a^	4.11^a^^,^ b	**0.003** *	0.65
	(4.79–6.12)	(3.98–5.35)	(3.43–4.80)		
Fore-aft GRF, 2nd peak (%BW)	−8.02	−8.11	−7.41	0.095	0.34
	(−8.76–−7.43)	(−8.91–−7.31)	(−8.13–−6.69)		
Hip position-initial contact	32.8	33.11	33.04	0.931	0.006
	(30.95–34.65)	(31.04–35.17)	(31.08–35)		
Max Hip Ext	3.06	2.62	2.5	0.210	0.12
	(2.71–3.42)	(2.18–3.05)	(1.97–3.04)		
Hip ROM	37.31	36.13	36.7	0.278	0.10
	(35.83–38.79)	(34.92–37.33)	(35.25–38.16)		
Knee position-initial contact	5.4	5.73	5.53	0.876	0.01
	(4.55–6.25)	(4.9–6.57)	(4.34–6.71)		
Max Knee Flex -stance	13.72	12.47	12.8	0.291	0.09
	(12.59–14.86)	(11.08–13.85)	(11.5–14.19)		
Max Knee Flex-swing	75.40	66.92^a^	70.81^a^^,^ b	**<0.001** *	0.60
	(73.21–77.57)	(64.77–69.08)	(68.7–72.93)		
Knee ROM	70.68	61.42^a^	58.25^a^	**<0.001** *	0.79
	(68.34–73.04)	(58.99–63.81)	(56.55–59.94)		
Ankle position-initial contact	−0.81	0.27^a^	−0.6^b^	**0.001** *	0.71
	(−1.21–−0.41)	(0.07–0.46)	(−0.93–−0.28)		
Max ankle PF-stance	−7.19	−5.89	−3.02^a^^,^ b	**<0.001** *	0.80
	(−8.3–−6.07)	(−6.98–−4.81)	(−3.73–−2.31)		
Max ankle DF-stance	14.49	15.11	14.67	0.556	0.04
	(13.34–15.63)	(14.24–15.98)	(13.93–15.41)		
Max ankle PF-swing	0.33	1.37^a^	1.13^a^	**<0.001** *	0.76
	(0.12–0.55)	(1.13–1.67)	(0.93–1.33)		
Ankle ROM	21.73	20.8	20.69	0.417	0.07
	(20.35–23.1)	(19.32–22.43)	(19.55–21.83)		

CI = Confidence interval; PF = plantar flexion; DF = dorsiflexion; Flex = flexion; Ext = extension; ROM = range of motion.

a Mean difference is significant at the 0.05 level compared with the Seal-In suspension.

b Mean difference is significant at the 0.05 level compared with the pin/lock suspension.

*shows significant differences among the three suspension systems.

### GDI

**Figure 3:**
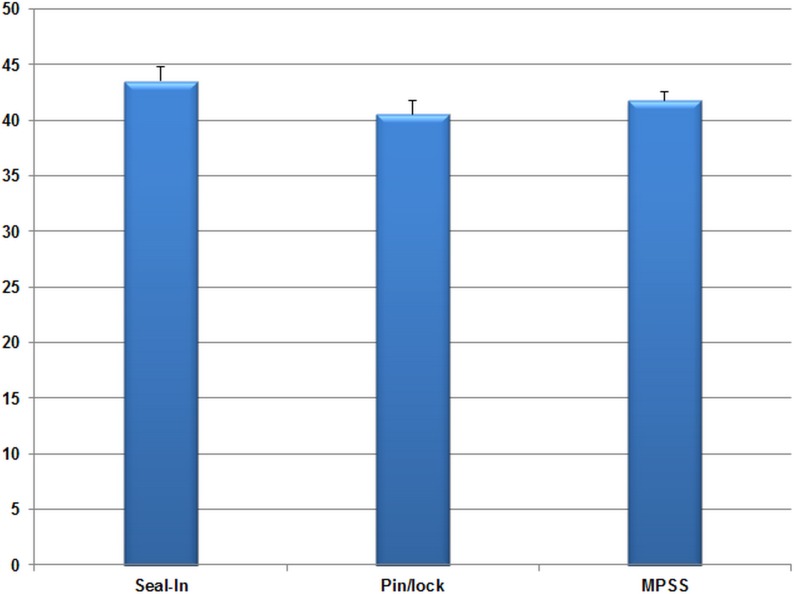
The comparison of GDI values among the suspension systems. Error bars show the standard error values.

The mean GDI for 13 subjects were 43.33, 40.57, and 39.87 with the Seal-In, pin/lock, and MPSS, respectively. Suspension type did not result in significant difference of the GDI values (*F*(2,24) = 2.11, *P* = 0.143, η_p_^2^ = 0.15). Figure 3 presents the comparison of mean GDI index values among the suspension systems.

## Discussion

The gait of lower limb amputees has long been studied to understand the kinematic and kinetic deviations resulting from the loss of ankle-foot (transtibial amputees) or knee-ankle-foot complex (transfemoral amputees). The effects of various prosthesis components on the gait of individuals with amputation have been investigated. Primarily, this study attempted to examine the effect of suspension type on walking kinetics and kinematics, pistoning and gait deviation with three different suspension systems. The previous research showed that the interface pressure with the suspension systems used in the current study were considerably different [16]. Thus, we hypothesized that gait characteristics would also be notably different among the MPSS, Seal-In, and pin/lock systems.

Transtibial amputees have different gait patterns from healthy individuals. As a result, the intact limb is said to undergo higher loading. To compensate, amputees adopt mechanisms, such as decreased walking speed, increased knee and hip moments and higher ankle ROM on the sound limb [2]. Based on the literature, the asymmetry in amputee gait reduces the time of stance [22]–[24] and the ground reaction forces [22], [25], [26] of the prosthetic limb compared with the sound limb.

Healthy individuals have a gait velocity of 1.2 m/s–1.5 m/s [27], [28]. No significant difference was observed in gait speed among the three suspension systems (*P* = 0.075). Also, previous studies revealed higher walking speed for transtibial amputees than our findings [15], [29], [30].

### Pistoning

Pistoning is used as a measure of suspension efficiency [31]. The findings in this study revealed that pistoning values were significantly different among the suspension systems during level walking both in the stance and swing phase with medium and large effect sizes of 0.69 and 0.79, respectively. The magnitudes of pistoning with the MPSS and pin/lock systems were compatible. The Seal-In system exhibited significantly lower pistoning during the swing phase compared with the pin/lock (2.0 vs. 4.9 mm, *P* = 0.002, η_p_^2^ = 0.57) and MPSS (2.0 vs. 3.3 mm, *P* = 0.002, η_p_^2^ = 0.57). The values were well-matched to those obtained during gait simulation in our previous study [17]; the gait simulation showed a pistoning range of 0 to 5.8 mm and the pistoning in the current study ranged between 0 to 5.1 mm.

### Ground Reaction Force

**Figure 4:**
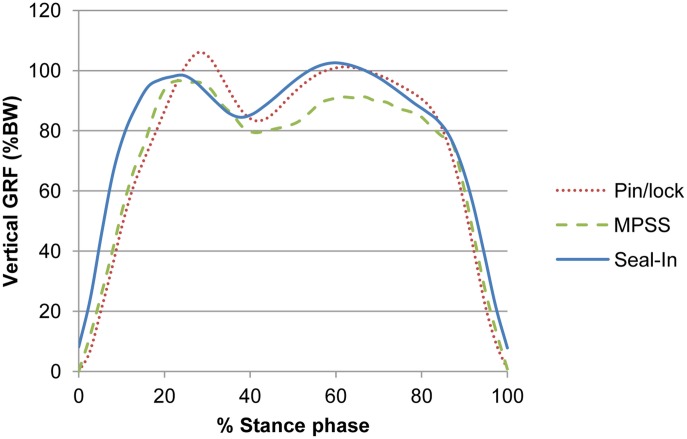
Vertical GRF for each suspension type. The vertical ground reaction force (GRF) pattern of the prosthetic limb for the three suspension systems.

The external forces exerted on the lower limbs during walking are defined as GRFs [32], [33]. The magnitude of peak GRF can determine level of shock absorption. All the suspension systems exhibited significant differences in the first peak of vertical GRF between the sound and prosthetic limbs. The sound limb exhibited significantly higher first peak vertical GRF compared with the prosthetic leg in the previous literature [30], [34], [35]. Our findings were consistent with those findings as the participants showed higher first peak value for the sound limb with all the systems (Table 2). Also, the suspension systems showed significantly different 1^st^ peak GRF values (*F(*2,24) = 13.01, *P = *0.000, η_p_^2^ = 0.52). High magnitude of first peak GRF indicates higher loading transferred to the limb joints. The MPSS showed lower values than the pin/lock (mean difference = 7.8; *P* = 0.006), which may indicate that lower external loading was applied to the joints (Figure 4).

Generally, there was significant difference between the suspension systems in the 2^nd^ peak of vertical GRF (*F*(2,24) = 18.80, *P* = 0.000, η_p_^2^ = 0.61). None of the suspension systems showed significant differences between the prosthetic and sound legs. Thus, it can be deduced that the dynamic foot used in this study (Talux) generated an added force during push off by storing energy and simulating the anatomical ankle plantar flexion. However, the magnitude of the second peak of vertical GRF was lower with the MPSS than the pin/lock (mean difference = 7.67). This result may be associated with the lower interface pressure within the prosthetic socket observed in the previous study [16].

The pattern of resultant fore-aft GRF revealed comparable acceleration forces for all the suspension systems (*F*(2,24) = 2.45, *P* = 0.107), and for both limbs. A larger deceleration force (braking force) was observed with the sound limb (*P* = 0.000 for all the systems), which conforms to the previous finding by Zmitrewicz et al. (2006) [36]. However, several minor differences in magnitudes are evident between the two studies, possibly due to the variations in prosthetic components, particularly the foot and walking velocity. The highest actual mean difference between the legs was seen with the MPSS (5.75). Braking peaks of prosthetic limb were lower with the MPSS than the pin/lock (*P* = 0.016, *d* = 0.78). This result possibly indicates better shock absorption with the MPSS. The duration of deceleration force was also dissimilar between the limbs, as the prosthetic side showed a larger value than the sound limb, which is compatible with the findings of Zmitrewicz et al. (2006) [36]. Propulsive force contributes to symmetrical gait pattern, balanced loading and steady walking speed. All the systems demonstrated similar magnitudes of propulsion force (for-aft GRF, 2^nd^ peak) for both limbs. This observation may reveal symmetry between the lower limbs.

### Spatiotemporal Parameters

Compared with the normal individuals, the amputee gait is characterized by lower velocity, greater swing time, longer step length, and increased cadence [28]. These characteristics are compensatory means of reducing instability and imbalance. In this study, cadence (number of steps per time unit) did not differ considerably between the sound and prosthetic legs for all suspension systems. However, the Seal-In system exhibited more homogenous cadence values between the legs (Table 2). The magnitudes were similar to the cadence values of other studies [27], [28].

Inconsistent step length is generally the result of uneven weight bearing through the lower limbs. Longer step length helps in relieving the load off the residual limb. There was no significant difference among the three systems (*F*(2,24 = 0.13, *P* = 0.817) and between the limbs. This was not consistent with the previous studies that showed significant difference in step length between the legs [29].

Prosthesis users tend to shift weight to the sound leg; consequently, the timing of prosthesis stance phase is lower [34]. Similarly, the stance phase was shorter with the prosthetic leg than the sound limb for all the suspension systems in our study (*d* = 1.3, 3.4 and 1.7 for the Seal-In, pin/lock and MPSS, respectively). The highest actual difference was seen with the pin/lock (66.7 vs. 61.7), while the lowest with the Seal-In (65.6 vs. 62.3) (Table 2). These results indicate that possibly the participants were more comfortable to walk with the Seal-In system, while probably the milking phenomenon resulted in pain and discomfort with the pin/lock suspension. Although statistically different, the actual differences might not be clinically relevant. The longer swing phase may be the result of the lighter prosthetic foot (carbon Talux) than the anatomical one [29].

### Kinematics

The previous literature on amputee’s gait biomechanics demonstrated slight deviations from the able-bodied gait pattern [28], [34]. Also, there are differences between the sound and amputated legs in unilateral amputees. In our study, the magnitudes of hip ROM were slightly higher with the sound leg than the prosthetic leg; however, the statistical analysis did not show any significance. Similarly, Bateni and Olney (2002) showed relative smaller ranges of hip angle for the amputated side [34]. There was no significant difference among the three systems on the prosthetic side (*P* = 0.240).

In the previous studies, less knee flexion was observed on the amputated side in comparison with the normal values in stance phase. Similarly, less knee flexion was seen in our study. This finding can be attributed to the inability of the prosthetic foot to produce the controlled plantar flexion as dorsiflexor eccentric contraction is missing [37]. Knee and foot motions are often synchronized. In most prosthetic feet, the ankle does not allow plantar flexion when weight is transferred to the toe section. If the knee at the amputated side is flexed to the mean normal value, excessive trunk lowering would produce an abnormal, inept gait [34]. However, the dynamic Talux foot allowed certain degrees of plantar flexion in this study.

Significant differences were seen in the maximum knee flexion on the prosthetic leg during the swing phase among the three suspension systems (*F(*2,24) = 18.40, *P* = 0.000, η_p_^2^ = 0.60). Significantly higher flexion was observed with the Seal-In system than the MPSS (*P* = 0.006). Also, the maximum knee flexion with the MPSS was higher than the pin/lock suspension (*P* = 0.041). The actual mean difference was higher between the Seal-In and pin/lock systems (8.48). The knee ROM was significantly higher on the prosthetic limb than the sound limb with all the systems and effect sizes were large (Table 2). The highest actual mean difference was seen with the Seal-In system (14.54), which may be clinically relevant as the knee ROM is important for foot clearance and demanding activities such as running. This finding is consistent with Colborne et al. (1992) [38]. The amputees often flex the amputated knee more than the sound knee to ensure foot clearance during the swing.

Gait progression is affected by the absence of anatomical ankle as more than 80% of mechanical power is generated by the plantar flexion in healthy individuals. The maximum ankle plantar flexion during the swing phase was significantly different among the systems (*F*(3,53) = 38.57, *P* = 0.000, η_p_^2^ = 0.76), and higher with the sound limb compared with the prosthetic limb with all the suspension systems (large effect sizes). The actual mean differences may be clinically relevant as the differences were high (more than 10°). Significant differences also existed in the ankle dorsiflexion in the stance phase between the sound and prosthetic limbs; the values were higher with the prosthetic leg (the actual mean differences were less than 8°). This can be attributed to the stiffness of prosthetic foot. The Talux foot has been reported to produce similar gait characteristics to the human foot [29]. Our participants also indicated that the Talux foot was more comfortable than their previous foot, particularly at heel strike and push off.

### GDI

Gait summary measures have been recently adopted as an index of gait deviations for various pathologies, such as cerebral palsy, Parkinson’s, and lower limb loss [20], [21], [39]. We adopted the GDI to investigate the possible gait deviation from the normal pattern with every suspension system. Kark et al. (2010) reported that the GDI is an appropriate measure for those with lower limb amputation [20]. They reported an average GDI of 84.2 (SD 9.4) for transtibial amputees. Nevertheless, our subjects showed GDI values from 39.87 to 43.33. The difference in findings may be attributed to the fact that Kark et al. (2010) did not consider hip rotation in their calculations. In our study, the Seal-In, MPSS and pin/lock were 5.54, 5.89, and 5.94 standard deviations away from the normal kinematics. There was no significant difference among the three suspension systems; only slight mean differences were seen. The previous studies showed high interface pressure and discomfort during walking with the Seal-In [16], [40]. In the current study, it showed the least deviation from the normal gait kinematics, which can be attributed to lower pistoning during gait reported in the former literature [17].

A previous study on the MPSS revealed higher satisfaction rates compared with the Seal-In and pin/lock suspension systems [17]. Lower peak pressure than the pin/lock suspension, particularly during the swing phase, has been also demonstrated [16]. Not surprisingly, the GDI scores revealed inferior gait kinematics than the normal individuals; yet, the three suspension systems exhibited similar clinical outcomes that enabled the amputees to ambulate. These findings need to be further investigated on amputees with different activity levels, and with various prosthetic feet. Moreover, the effect of parameters such as the residual limb length, volume, cause of amputation, skin conditions can be further studied on the gait pattern with various suspension systems. Although, the main differences among the suspension types had high effect sizes, larger sample size may provide stronger evidence for the current findings. It is likely that those parameters that showed no difference exhibit significance if tested on higher number of amputees.

While it is common to observe significant differences between the sound and prosthetic limbs in amputees, non-significance may be considered as positive effect of prosthetic components. On the other hand, several kinetic and kinematic parameters did not show high actual mean differences among the suspension systems in this study. The main differences with high effect sizes were seen for the 2^nd^ peak of vertical GRF and the knee range of motion between the Seal-In and MPSS (10.94 and 12.43, respectively). In summary, it may be concluded from the overall findings that the new prosthetic suspension system (MPSS) can be used clinically as an alternative suspension system for lower limb amputees.

## Conclusions

Gait biomechanics was significantly influenced by the suspension type. Main differences between the suspension systems were evident in the GRF (vertical and fore-aft), knee and ankle angles; yet, not all of them are considered clinically relevant. Most specifically, the ankle angles are mainly influenced by the type of prosthetic foot, not the suspension system. The MPSS may reduce the loading over the proximal limb joints compared with the pin/lock system. Pistoning was also significantly altered by the types of suspension system. The Seal-In liner was the most effective suspension system in reducing the vertical movement during level walking. We should emphasize that prosthetic foot characteristics and alignment will also influence the gait pattern in addition to the suspension system. This study is hoped to enhance the knowledge of clinicians on gait biomechanics with various available suspension systems.

## Supporting Information

Protocol S1 Trial protocol.
(PDF)

## References

[1] Macfarlane PA, Nielsen DH, Shurr DG, Meier K (1991) Gait Comparisons for Below-Knee Amputees Using a Flex-Foot (TM) Versus a Conventional Prosthetic Foot. JPO: J Prosthet Orthot 3: 150–161.

[2] Nolan L, Lees A (2000) The functional demands on the intact limb during walking for active trans-femoral and trans-tibial amputees. Prosthet Orthot Int 24: 117–125. 11061198 10.1080/03093640008726534

[3] Lemaire ED, Fisher FR (1994) Osteoarthritis and elderly amputee gait. Arch Phys Med Rehabil 75: 1094. 7944914 10.1016/0003-9993(94)90084-1

[4] Melzer I, Yekutiel M, Sukenik S (2001) Comparative study of osteoarthritis of the contralateral knee joint of male amputees who do and do not play volleyball. J Rheumatol 28: 169–172. 11196520

[5] Silverman AK, Fey NP, Portillo A, Walden JG, Bosker G, et al (2008) Compensatory mechanisms in below-knee amputee gait in response to increasing steady-state walking speeds. Gait Posture 28: 602–609. 18514526 10.1016/j.gaitpost.2008.04.005

[6] Winter DA, Sienko SE (1988) Biomechanics of below-knee amputee gait. J Biomech 21: 361–367. 3417688 10.1016/0021-9290(88)90142-x

[7] Goujon H, Bonnet X, Sautreuil P, Maurisset M, Darmon L, et al (2006) A functional evaluation of prosthetic foot kinematics during lower-limb amputee gait. Prosthet Orthot Int 30: 213–223. 16990231 10.1080/03093640600805134

[8] Schmalz T, Blumentritt S, Jarasch R (2002) Energy expenditure and biomechanical characteristics of lower limb amputee gait:-The influence of prosthetic alignment and different prosthetic components. Gait Posture 16: 255–263. 12443950 10.1016/s0966-6362(02)00008-5

[9] Torburn L, Perry J, Ayyappa E, Shanfield SL (1990) Below-knee amputee gait with dynamic elastic response prosthetic feet: a pilot study. J Rehabil Res Dev 27: 369–384. 2089148 10.1682/jrrd.1990.10.0369

[10] Van der Linden M, Solomonidis S, Spence W, Li N, Paul J (1999) A methodology for studying the effects of various types of prosthetic feet on the biomechanics of trans-femoral amputee gait. J Biomech 32: 877–889. 10460124 10.1016/s0021-9290(99)00086-x

[11] Hachisuka K, Dozono K, Ogata H, Ohmine S, Shitama H, et al (1998) Total surface bearing below-knee prosthesis: advantages, disadvantages, and clinical implications. Arch Phys Med Rehabil 79: 783–789. 9685091 10.1016/s0003-9993(98)90356-2

[12] Kristinsson Ö (1993) The ICEROSS concept: a discussion of a philosophy. Prosthet Orthot Int 17: 49–55. 8337100 10.3109/03093649309164354

[13] Narita H, Yokogushi K, Shi S, Kakizawa M, Nosaka T (1997) Suspension effect and dynamic evaluation of the total surface bearing (TSB) trans-tibial prosthesis: a comparison with the patellar tendon bearing (PTB) trans-tibial prosthesis. Prosthet Orthot Int 21: 175–178. 9453088 10.3109/03093649709164551

[14] Yigiter K, Sener G, Bayar K (2002) Comparison of the effects of patellar tendon bearing and total surface bearing sockets on prosthetic fitting and rehabilitation. Prosthet Orthot Int 26: 206–212. 12562067 10.1080/03093640208726649

[15] Boutwell E, Stine R, Hansen A, Tucker K, Gard S (2012) Effect of prosthetic gel liner thickness on gait biomechanics and pressure distribution within the transtibial socket. J Rehabil Res Dev 49: 227–240. 22773525 10.1682/jrrd.2010.06.0121

[16] Eshraghi A, Abu Osman NA, Gholizadeh H, Ali S, Sævarsson SK, et al (2013) An experimental study of the interface pressure profile during level walking of a new suspension system for lower limb amputees. Clin Biomech 28: 55–60. 10.1016/j.clinbiomech.2012.10.002 23157843

[17] Eshraghi A, Abu Osman NA, Karimi MT, Gholizadeh H, Ali S, et al (2012) Quantitative and Qualitative Comparison of a New Prosthetic Suspension System with Two Existing Suspension Systems for Lower Limb Amputees. Am J Phys Med Rehabil 91: 1028–1038. 23168378 10.1097/PHM.0b013e318269d82a

[18] Gholizadeh H, Abu Osman NA, Kamyab M, Eshraghi A, Wan Abas WA, et al (2012) Transtibial prosthetic socket pistoning: static evaluation of Seal-In X5 and Dermo liner using motion analysis system. Clin Biomech 27: 34–39. 10.1016/j.clinbiomech.2011.07.004 21794965

[19] Farahmand F, Rezaeian T, Narimani R, Dinan H (2006) Kinematic and Dynamic Analysis of the gait cycle of above knee amputees. Scientia Iranica 13: 261–271.

[20] Kark L, Vickers D, McIntosh A, Simmons A (2012) Use of gait summary measures with lower limb amputees. Gait Posture 35: 238–243. 22000790 10.1016/j.gaitpost.2011.09.013

[21] Schwartz MH, Rozumalski A (2008) The gait deviation index: a new comprehensive index of gait pathology. Gait Posture 28: 351–357. 18565753 10.1016/j.gaitpost.2008.05.001

[22] Baker P, Hewison S (1990) Gait recovery pattern of unilateral lower limb amputees during rehabilitation. Prosthet Orthot Int 14: 80–84. 2235305 10.3109/03093649009080327

[23] Breakey J (1976) Gait of unilateral below-knee amputees. Orthot Prosthet 30: 17–24.

[24] Cheung C, Wall J, Zelin S (1983) A microcomputer-based system for measuring temporal asymmetry in amputee gait. Prosthet Orthot Int 7: 131–140. 6647009 10.3109/03093648309166585

[25] Dingwell J, Davis B, Frazder D (1996) Use of an instrumented treadmill for real-time gait symmetry evaluation and feedback in normal and trans-tibial amputee subjects. Prosthet Orthot Int 20: 101–110. 8876003 10.3109/03093649609164426

[26] Skinner HB, Effeney DJ (1985) Gait analysis in amputees. Am J Phys Med Rehabil 64: 82–89. 3887934

[27] Isakov E, Keren O, Benjuya N (2000) Trans–tibial amputee gait: time–distance parameters and EMG activity. Prosthet Orthot Int 24: 216–220. 11195356 10.1080/03093640008726550

[28] Winter DA (1991) Biomechanics and motor control of human gait: normal, elderly and pathological. Waterloo: University of Waterloo Press.

[29] Supan T, Lebiedowska M, Dodson R, Verhulst S, Dufour M (2010) The Effect of a Talux (R) Prosthetic Foot on Gait Parameters and Limb Loading of Nonvascular Transtibial Amputees. JPO: J Prosthet Orthot 22: 43–52.

[30] Vanicek N, Strike S, McNaughton L, Polman R (2009) Gait patterns in transtibial amputee fallers vs. non-fallers: Biomechanical differences during level walking. Gait Posture 29: 415–420. 19071021 10.1016/j.gaitpost.2008.10.062

[31] Eshraghi A, Abu Osman NA, Gholizadeh H, Karimi M, Ali S (2012) Pistoning assessment in lower limb prosthetic sockets. Prosthet Orthot Int 36: 15–24. 22269941 10.1177/0309364611431625

[32] Engsberg J, Lee A, Tedford K, Harder J (1993) Normative ground reaction force data for able-bodied and trans-tibial amputee children during running. Prosthet Orthot Int 17: 83–89. 8233773 10.3109/03093649309164361

[33] Stergiou N, Giakas G, Byrne JE, Pomeroy V (2002) Frequency domain characteristics of ground reaction forces during walking of young and elderly females. Clin Biomech 17: 615–617. 10.1016/s0268-0033(02)00072-4 12243722

[34] Bateni H, Olney SJ (2002) Kinematic and kinetic variations of below-knee amputee gait. JPO: J Prosthet Orthot 14: 2–10.

[35] Gailey R, Allen K, Castles J, Kucharik J, Roeder M (2008) Review of secondary physical conditions associated with lower-limb amputation and long-term prosthesis use. J Rehabil Res Dev 45: 15. 18566923 10.1682/jrrd.2006.11.0147

[36] Zmitrewicz RJ, Neptune RR, Walden JG, Rogers WE, Bosker GW (2006) The effect of foot and ankle prosthetic components on braking and propulsive impulses during transtibial amputee gait. Arch Phys Med Rehabil 87: 1334–1339. 17023242 10.1016/j.apmr.2006.06.013

[37] Smidt GL (1990) Gait in rehabilitation: Churchill Livingstone.

[38] Colborne GR, Naumann S, Longmuir PE, Berbrayer D (1992) Analysis of mechanical and metabolic factors in the gait of congenital below knee amputees: A comparison of the SACH and Seattle feet. Am J Phys Med Rehabil 71: 272–278. 1388973 10.1097/00002060-199210000-00004

[39] Cimolin V, Galli M, Vimercati SL, Albertini G (2011) Use of the Gait Deviation Index for the assessment of gastrocnemius fascia lengthening in children with Cerebral Palsy. Res Dev Disabil 32: 377–381. 21075594 10.1016/j.ridd.2010.10.017

[40] Ali S, Abu Osman NA, Mortaza N, Eshraghi A, Gholizadeh H, et al (2012) Clinical investigation of the interface pressure in the trans-tibial socket with Dermo and Seal-In X5 liner during walking and their effect on patient satisfaction. Clin Biomech 27: 943–948. 10.1016/j.clinbiomech.2012.06.004 22795863

