# Supplementary material for: Gait Biomechanics of Individuals with Transtibial Amputation: Effect of Suspension System
Source: PLoS One. 2014 May 27;9(5):e96988. doi: 10.1371/journal.pone.0096988 (PMC4035274; doi:10.1371/journal.pone.0096988)
Supplement: Protocol S1 — Trial protocol. (PDF) [file pone.0096988.s001.pdf]

UNIVERSITI MALAYA
PUSAT PERUBATAN UM

JAWATANKUASA ETIKA PERUBATAN
PUSAT PERUBATAN UNIVERSITI MALAYA
ALAMAT: LEMBAH PANTAI, 59100 KUALA LUMPUR, MALAYSIA
TELEFON: 03-79493209 FAKSIMILI: 03-79494638

Permohonan Menjalankan Projek Penyelidikan
(Application to Conduct Research Project)

1. Tajuk projek *(Project title):*
   Design, development and clinical evaluation of a new prosthetic suspension system for lower limb amputees

2. Penyiasat utama (*Principal investigator*):
   2.1 Nama *(Name)* : AREZOO ESHRAGHI

   2.2 Jawatan *(Designation)* : PhD STUDENT

   2.3 Jabatan *(Department)* : Biomedical Engineering

   2.4 E-mel *(E–mail)* : arezoo@um.edu.my

   Sila lampirkan 'curriculum vitae' yang ringkas *(Please attach your brief CV)*

3. Penyiasat-penyiasat lain *(Other investigators):*

	Nama *(Name)*	Jawatan & Jabatan *(Designation & Department)*	Tandatangan/Cop *(Signature & Stamp)*
3.1	Assoc. Prof. Dr. Noor Azuan Abu Osman	Supervisor, Deputy Dean, Dept Of Biomedical engineering Faculty of Engineering	Assoc. Prof. Dr. Noor Azuan Abu Osman
3.2	Dr. Chung TZE YANG	Rehabilitation Physician Department of Rehabilitation Medicine Faculty of Medicine	Dr. Chung TZE YANG

4. Keadaan projek, jika berkenaan (*Project status, if applicable):*

   4.1 Projek ini adalah projek *(This project is)* ■ Baru *(New)* ☐ Sambungan *(Continuation)*

   4.2 Jika sambungan, sila beri butir-butir berikut:
   (If it is a continuation project, please give details as follows) :

   a. Tajuk asas projek *(jika berlainan)* :
      (Initial project title [if applicable])

   b. Tarikh bermula (Date of commencement) :

   c. Tarikh akan siap *(Date of completion)*:

   d. Bantuan diterima dahulu dari *(Previous sponsorship)*

5 . Nyatakan jika projek ini adalah sebahagian keperluan pengajaran ijazah/lepasan ijazah
(Please state whether this project is for fulfilment of basic/postgraduate degree)

5.1 ■ Ya/*Yes*
(Nyatakan pengkhususan ijazah, samada Sarjana Muda, Sarjana atau PhD / *State name of degree, e.g Bachelor, Master or PhD)*
PhD

5.2 □ Tidak/*No*

6. Geran untuk kajian (Nyatakan sumber geran) / *Research funding (State source of funding)*

6.1 □ Tiada geran / *No funding* □ Tiada geran diperlukan / *No funding needed*

□ Untuk dimohon kemudian / *To apply later*

6.2 □ Penaja kajian / *Trial sponsor* - Nyatakan nama penaja / *State name of sponsor*

____

6.3 ■ Geran kajian / *Research fund* – Nama geran kajian / *Name of fund/research vote*

________FRGS____________

7. Maklumat projek / *Project information*

7.1 Latar belakang, bahan-bahan rujukan dan rasional yang berkaitan dengan projek ini
(Background, literature reference and rationale for this project)

Amputation is a complex problem for patients, the health care system, and the country. Dysvascular disease associated with diabetes accounts for about 82% of all limb losses. More than 180 million patients suffer from diabetes according to data from the WHO, of which Malaysia alone will face 2.48 million in 2030 that is 164% increase compared to year 2000 [1].

Safe and effective use of prosthetic limbs requires that the prosthesis be suspended consistently and comfortably on the limb during patient activity. Selection of the optimal suspension is paramount to achieving efficient and safe prosthetic ambulation [2]. An improperly fitting suspension may result in discomfort, pistoning of the device around the residual limb, skin breakdown, increased energy consumption, gait deviations, and falls. A good suspension reduces the risk of skin breakdown or irritation by minimizing the limb movement (pistoning) inside the prosthesis [3]. The research should develop a new prosthetic suspension system to address some of the shortcomings of current designs.

Many choices in suspension system are available, and clinicians often rely on personal intuition and experience to

choose which system is appropriate for which patient [4]. Clinicians, administrators, medical researchers, and third-party payers are required to make decisions about the quality of care and the effectiveness of the prosthesis. The use of silicone liners in prosthetics is not new. It has two main functions, namely, protection of the amputation stump and suspension of the prosthesis [5, 6]. Suspension in silicone suction socket can be achieved in different ways, such as shuttle lock, sleeve, or a Hypobaric Sealing Membrane (HSM) around the liner (a new technology in silicone liners).

On the basis of the researcher's experience, available suspension systems for lower limb amputations have not yet fully addressed the patients' needs. A great number of amputees have some problems in their stump, like contracture, diabetic, or skin problem and they complain about the suspension system in terms of donning and doffing, gait and pain. It was the starting point to deal with this topic in general and invent a new suspension system which can cover some of the shortcomings of the existing suspension systems for lower limb amputees.

REFERENCES

1. Wild S, Gojka, Green A, Sicree R and King H. Global Prevalence of Diabetes: Estimates for the year 2000 and projections for 2030. *Diabetes Care* 2004; 27(5): 1047-1054.
2. Klute G, Glaister B, Berge J. Prosthetic liners for lower limb amputees: a review of the literature. *Prosthet Orthot Int* 2010; (00): 1-8.
3. Kristinsson Ö. The ICEROSS concept: a discussion of a philosophy. *Prosthet Orthot Int* 1993; 17(1):49-55.
4. Datta D, Vaidya S, Howitt J, Gopalan L. Outcome of fitting an ICEROSS prosthesis: views of trans-tibial amputees. *Prosthet Orthot Int* 1996; 20(2):111-115.
5. E.C. T. Baars & J. H. B. Geertzen, Literature review of the possible advantages of silicon liner socket use in trans-tibial prostheses, *Prosthet Orthot Int* 2005; 29(1): 27 – 37
6. E. C. T. Baars, P. U. Dijkstra, & J. H. B. Geertzen, Skin problems of the stump and hand function in lower limb amputees: A historic cohort study, *Prosthet Orthot Int*, June 2008, 32(2): 179-185

7.2 Tujuan-tujuan projek *(Objectives of the Project)*

This study embarks on the following objectives:

- To design and fabricate a new prosthetic suspension system.
- To obtain kinematics and kinetics of trans-tibial and trans-femoral amputees gait using the new suspension system and compare that to the locking liner and Seal-in liner by using motion analysis approach.
- To evaluate the biomechanical characteristics of each of the suspension systems (new system, locking or seal-in) in terms of comfort, function and satisfaction in amputees.
- To evaluate prosthesis users' satisfaction and perceived problems with the three suspension systems, including the new system.

7.3 Keputusan-keputusan yang dianggapkan, jika ada *(Expected outcome, if any)*

The project will result in the development of a new prosthetic suspension system which is expected to be cheaper, result in low levels of pistoning, improve the amputee's satisfaction, and cause less pressure at the stump-liner-socket interface.

7.4 Kepentingan dan/atau kegunaan lanjut, jika ada *(Significance and/or further practical applications, if any).*

The new prosthetic suspension system has the potential to be used as suspension system for both transtibial and transfemoral prostheses.

7.5 Jadual waktu bagi projek ini, termasuk tarikh bermula, fasa-fasa dalamnya dan tarikh selesai
(Time frame for project, including duration, phases, start date and end date estimates)

Items	2010			2011				2012				2013		
	Apr'10	Jul'10	Oct'10	Jan'11	Apr'11	Jul'11	Oct'11	Jan'12	Apr'12	Jul'12	Oct'12	Jan'13	Apr'13	Jul'13
Literature review														
Selection and evaluation of patients														
Design and fabrication of prostheses<br>Adjust alignment and fitness of prostheses														
Evaluation of prostheses and gait training														
Evaluation of gait (kinematics – kinetics) in motion lab														
Prosthetics questionnaire evaluation (satisfaction)														
Evaluation of interface pressure														
Data analysis														
Assessment and reports														

8. Methodologi/kaedah yang dicadangkan / *Proposed methodology*

8.1 Rekabentuk projek (Sila sertakan nota keterangan/apendiks jika perlu)
Study design (Please submit explanatory notes/appendices if necessary)

Sila beri butir-butir mengenai populasi atau bahan-bahan yang akan disiasat, prosedur-prosedur, cara-cara memproses data dan sebagainya. *(Please give details concerning population or materials to be studied, procedures, and data processing methods etc)*

Following a review of literature, a new magnetic suspension system will be designed, fabricated and fine-tuned. The prototype will be tested mechanically under loading. The finite element analysis is then performed to study the possible loading behaviors of the system during ambulation.
The result of the finite element analysis and mechanical testing will be used to modify and enhance the model. Next, a pilot study will be conducted on transtibial and trasfemoral amputees.
Following the design adjustments, lower limb amputees (transtibial and transfemoral) will be selected for clinical evaluation. The inclusion criteria would be:

- no medical contraindications for engaging in physical activities
- no ulcer or open wound in the limb
- old prosthetic user (more than 1 month)
- no upper limb weakness or disability

The demographic and physical data will be collected following informed consent. Stump characteristics will be recorded as well.
Three pair of prostheses will be fabricated for each subject using: new prototype, pin & lock system, and seal-in liner. All prostheses will be made by the researcher herself to ensure consistent manufacture, fit and alignment.
Each prosthesis consists of a total surface weight bearing socket, silicone liner, shuttle lock, valve or new magnetic system, tube adaptor, clamp adaptor, and foot. The components would be as follows (in addition to the new prototype):

- Iceross Seal-In® X5 Transtibial Liner
- Icelock Expulsion Valve 551
- 4-prong socket Adapter
- 3-prong socket adapter
- Male Pyramid Insert for Prong
- Female Pyramid Tube Clamp
- Female Pylon
- Talux Foot (transtibial)
- Iceross Dermo Locking liner

In addition, the below components will be utilized for transfemoral amputees:
3R60 hydraulic knee joint (Otto Bock)
SACH foot

Subjects will be required to walk with their prostheses in the Brace and Limb Laboratory (Clinical P&O Lab) and do various physical activities, i.e. donning and doffing of prosthesis, stand up, sit down, standing, sitting, walking and running. Next, they will wear each pair of prostheses for one month and a modified PEQ questionnaire will be employed to assess the user's satisfaction.

Gait analysis will be performed by a Vicon motion capture system (seven MX-F20 cameras, 50HZ) synchronized with two Kistler and two AMTI force plates integrated into Vicon Nexus 1.4 software to record the data. Helen Hayes marker set will be used. The positions of most of the anatomical landmarks will be assumed to coincide with the center of the marker used. The force platform signals are filtered with a Butterworth low pass filter at 10Hz. For each trial, angular displacements, internal joint moments, joint powers, and the force applied on the limb will be plotted over one gait cycle. Each subject will complete five successful trials using each of the suspension systems randomly. Pistoning movements inside the prosthetic socket will be measured for each subject during walking, stair and ramp negotiation. The data will be used to study the shear and stress forces at the socket-liner- limb interface by finite element analysis (FEA).

Socket-liner interface pressure will be determined by F-scan socket sensors (Tekscan).

All the above mentioned experiments will be done by 2 conventional and our new suspension systems.

8.2 Teknik pengumpulan data yang digunakan / *Data collection technique involved:*

- ■ Prospektif / *Prospective*
- ☐ Retrospektif / *Retrospective*
- ☐ Lain-lain (Sila nyatakan) / *Other technique (Please specify)* :

____

8.3 Adalah subjek bernyawa terlibat? / *Are human subjects involved?*

■ Ya / *Yes*

Sila pilih / *Please Select*:

- ☐ Pesakit/*Patients* (Sila nyatakan jumlah pesakit yang terlibat/*Please state number of patients involved*)

  ___________10 trans-tibial, 10 trans-femoral_________

- ☐ Sukarelawan/*Volunteers or Normal controls*
- ☐ Kaji selidik kumpulan sasaran (Sila lampirkan Borang Kaji Selidik) / *Survey of target groups (Please attach questionnaire)*

☐ Tidak / *No*

8.4 Adakah terdapat kaedah terapeutik/dadah baru diuji? / *Are new therapeutic procedures/drugs being tested?*

☐ Ya / *Yes*
Nyatakan prosedur/dadah yang digunakan (*State the procedures/drugs used)*

■ Tidak / *No*

8.5 Adakah prosedur yang mengganggu terlibat /*Are invasive procedure applied?*

☐ Ya/ Yes
Sila nyatakan prosedur / State the procedures

■ Tidak / No

8.6 Adakah terdapat sumber/peralatan yang akan digunakan di FPUM atau PPUM? Jika ya, sila senaraikan
(*Will resources/ equipment in FOM or UMMC be utilized? If yes, please state)*

☐ Ya / *Yes*

■ Tidak / *No*

9. Isu Etika / *Ethical Issues*

9.1 Adakah projek ini mematuhi prinsip-prinsip "Declaration of Helsinki"/garis panduan Malaysian Good Clinical Practice (GCP)
Does this project conform to the Declaration of Helsinki/Malaysian Good Clinical Practice (GCP) Guidelines?

■ Ya / *Yes*

☐ Tidak / *No*

9.2 Apakah faedah kepada subjek di dalam projek ini?
(*What are the benefits to research subjects of this study?)*

There is no direct benefit to the research subjects.

9.3 Apakah risiko kepada subjek di dalam projek ini?
(*What are the risks to the research subjects of this study?)*

There is no known risk to the research subjects.

9.4 Bagaimanakah perlindungan untuk Serious Adverse Events (SAEs) (contoh: insurans, pembayaran, dll) disediakan?
(*How is coverage for Serious Adverse Events (SAEs) (eg: insurance, payment, etc) provided for?)*

Not Applicable

9.5. Adakah rawatan bermanfaat masih diberikan kepada subjek selepas projek ini selesai? Jika tidak, sila nyatakan sebab.
(Will beneficial treatment still be provided for research subjects after completion of the study? If no, please state why)

☐ Ya / *Yes*

■ Tidak / *No*

______ Not Applicable__________

9.6 Adakah maklumat dan keizinan pesakit akan disediakan? / *Will information to the patient and informed consent be provided?*

- [x] Ya (sila isikan Borang Maklumat Pesakit dan Borang Keizinan Pesakit yang disediakan)
  Yes (please fill in **Patient Information Sheet** and **Consent Form** *provided)*

- [ ] Tidak / *No*

9.7 Adakah perbelanjaan projek ini dikenakan kepada subjek? Jika ya, sila nyatakan jumlah perbelanjaan yang terlibat. Jika tidak, siapakah yang akan menanggung perbelanjaan bagi kajian ini?
(Are expenses borne by the research subjects? If yes, please state the expenses incurred. If no, how are expenses provided for?)

- [ ] Ya/*Yes*

Expenses will be subsidized by Department of Biomedical Engineering, Faculty of engineering, University of Malaya.

- [x] Tidak/*No*

9.8 Adakah terdapat sebarang bentuk bayaran ditawarkan kepada subjek? Jika ada, berapakah jumlahnya?
(Will any form of payment be offered to research subjects? If yes, what is the amount?)

■ Ya/*Yes*

50 RM per session for the travel and snacks as honorarium.

☐ Tidak/*No*

10. Butir-butir lain *(Other information)*

11. Ulasan Dan Kebenaran Ketua Jabatan/Unit/Makmal Terlibat (*Comments and permission of relevant Head of Department/Laboratory)*

**Sila tandakan [✓] *(Please tick [✓])*:**	**Tandatangan & Cop/Tarikh *(Signature & Stamp/Date)***
☐ Jabatan Anatomi *(Department of Anatomy)*	
☐ Jabatan Anestesiologi *(Department of Anaesthesiology)*	
☐ Jabatan Farmakologi *(Department of Pharmacology)*	
☐ Jabatan Farmasi, FPUM *(Department of Pharmacy, FOM)*	
☐ Jabatan Farmasi, PPUM *(Department of Pharmacy, UMMC)*	
☐ Jabatan Fisiologi *(Department of Physiology)*	
☐ Jabatan Oftalmologi *(Department of Ophthalmology)*	
☐ Jabatan Obstetrik & Ginekologi *(Department of Obstetrics & Gynaecology)*	
☐ Jabatan Otorhinolaringologi *(Department of Otorhinolaryngology)*	
☐ Jabatan Parasitologi *(Department of Parasitology)*	
☐ Jabatan Patologi *(Department of Pathology)*	
☐ Jabatan Pediatrik *(Department of Paediatrics)*	

- [ ] Jabatan Pengimejan Bio-perubatan
  (Department of Biomeical Imaging)

- [ ] Jabatan Perubatan
  (Department of Medicine)

- [ ] Jabatan Perubatan Kemasyarakatan dan Pencegahan
  (Department of Social and Preventive Medicine

- [ ] Jabatan Perubatan Mikrobiologi
  Department of Medical Microbiology

- [ ] Jabatan Perubatan Molekular
  (Department of Molecular Medicine)

- [x] Jabatan Perubatan Pemulihan
  (Department of Rehabilitation Medicine)

- [ ] Jabatan Perubatan Psikologi
  (Department of Psychological Medicine)

- [ ] Jabatan Perubatan Rawatan Utama
  (Department of Primary Care Medicine)

- [ ] Jabatan Perubatan Transfusi
  Department of Tranfusion Medicine

- [ ] Jabatan Sains Kejururawatan
  (Department of Nursing Science)

- [ ] Jabatan Surgeri Ortopedik
  (Department of Orthopaedic Surgery)

- [ ] Jabatan Surgeri
  Department of Surgery

- [ ] Jabatan Trauma & Kecemasan
  Department of Trauma & Emergency

- [ ] Unit Onkologi Klinikal, PPUM
  (Clinical Oncology Unit, UMMC)

- [ ] Unit Perubatan Sukan
  Sports Medicine Unit

- [ ] Lain-lain - Sila nyatakan
  (Others – Please specify)

12. Tandatangan Pemohon *(Signature of Applicant)*

_____________2 Mar 2012_______
Tarikh (*Date)*

__ AREZOO ESHRAGHI _______________
Tandatangan *(Signature)*

________AREZOO ESHRAGHI____
Nama Penuh (*Name in Full)*

13. Komen-komen Ketua Jabatan Pemohon *(Comments of Head of Department)*

Projek ini disokong/Tidak disokong *(Support/Not supported)*

Supported

----2 March 2012------------------
Tarikh (*Date)*

_____________Dr. Ahmad Khairi___
Tandatangan & Cop Ketua Jabatan
(*Signature & Stamp of Head of Deparment)*
